# Efficacies of omadacycline + amikacin + imipenem and an all-oral regimen omadacycline + clofazimine + linezolid in a mouse model of *M. abscessus* lung disease

**DOI:** 10.1128/msphere.00381-24

**Published:** 2024-07-09

**Authors:** Elisa H. Ignatius, Binayak Rimal, Chandra M. Panthi, Daniel C. Belz, Christopher K. Lippincott, Daniel H. Deck, Alisa W. Serio, Gyanu Lamichhane

**Affiliations:** 1 Division of Infectious Diseases, Department of Medicine, School of Medicine, Johns Hopkins University, Baltimore, Maryland, USA; 2 Center for Nontuberculous Mycobacteria and Bronchiectasis, School of Medicine, Johns Hopkins University, Baltimore, Maryland, USA; 3 Division of Clinical Pharmacology, Department of Medicine, School of Medicine, Johns Hopkins University, Baltimore, Maryland, USA; 4 Division of Pulmonary and Critical Care Medicine, Department of Medicine, School of Medicine, Johns Hopkins University, Baltimore, Maryland, USA; 5 Paratek Pharmaceuticals Inc., King of Prussia, Pennsylvania, USA; University of Nebraska Medical Center College of Medicine, Omaha, Nebraska, USA

**Keywords:** *M. abscessus*, omadacycline, amikacin, clofazimine, imipenem, linezolid

## Abstract

Treatment outcomes for *Mycobacteroides abscessus* (*Mab*, also known as *Mycobacterium abscessus*) disease are still unsatisfactory, mainly due to issues with drug toxicity, tolerability, and efficacy. Treating *Mab* disease is challenging due to its high baseline antibiotic resistance, initial requirement for intravenous therapy, and poor medication tolerance. Omadacycline, a new tetracycline, is active against *Mab*. Since any new antibiotic effective against *Mab* is expected to be used in combination with other antibiotics, we evaluated the efficacy of two triple-drug combinations comprising omadacycline, omadacycline + amikacin + imipenem, and omadacycline + clofazimine + linezolid against two contemporary *Mab* clinical isolates in a mouse model of *Mab* lung disease. Antibiotic administration was initiated 1-week post-infection and was given daily, with *Mab* burden in the lungs at treatment completion serving as the endpoint. Omadacycline alone moderately reduced *Mab* levels and maintained better health in mice compared to untreated ones, which typically suffered from the infection. The omadacycline + clofazimine + linezolid combination showed immediate bactericidal activity and enhanced efficacy over 6 weeks, particularly against the more resistant strain (M9507). However, the clofazimine + linezolid combination lacked early bactericidal activity. When combined with amikacin and imipenem, omadacycline did not improve the regimen’s effectiveness over 4 weeks of treatment. Our study showed that omadacycline + clofazimine + linezolid exhibited significant bactericidal activity over an extended treatment duration. However, adding omadacycline to amikacin and imipenem did not improve regimen effectiveness against the evaluated clinical isolates within 4 weeks. Further research in *Mab* disease patients is needed to determine the most effective omadacycline-containing regimen.

**IMPORTANCE:**

*Mycobacteroides abscessus* is a common environmental bacterium that causes infections in people with compromised lung function, including those with bronchiectasis, cystic fibrosis, chronic obstructive pulmonary disease, and weakened immune systems, especially among older individuals. Treating *M. abscessus* disease is challenging due to the limited effectiveness and toxicity of current antibiotics, which often require prolonged use. Omadacycline, a new antibiotic, shows promise against *M. abscessus*. Using a mouse model that mimics *M. abscessus* disease in humans, we studied the effectiveness of including omadacycline with recommended antibiotics. Adding omadacycline to clofazimine and linezolid significantly improved treatment outcomes, rapidly clearing the bacteria from the lungs and maintaining effectiveness throughout. This oral combination is convenient for patients. However, adding omadacycline to amikacin and imipenem did not improve treatment effectiveness within 4 weeks. Further study with *M. abscessus* patients is necessary to optimize omadacycline-based treatment strategies for this disease.

## INTRODUCTION

Mycobacterial diseases, by virtue of various resistance mechanisms in the etiological mycobacterium and the presence of persister subpopulations, require prolonged courses of multidrug therapy (1, 2). While all-oral regimens have become the standard of care for even extensively drug-resistant tuberculosis, the treatment of non-tuberculous mycobacteria (NTM), especially rapid grower species such as *Mycobacteroides abscessus* (*Mab*, also known as *Mycobacterium abscessus*), often still requires intravenous (IV) therapy (3–5). These IV agents typically require multiple daily doses, necessitate placement and maintenance of parenteral access, and are often limited in duration and dose due to their associated toxicities and intolerances. Notably, there are no FDA-approved treatments for *Mab* disease, and current recommendations, which involve repurposed antibiotics, yield cure rates of only 30%–50% (6, 7). There is now increasing momentum toward developing and studying novel drugs or drug combinations for treating *Mab* disease, evidenced by the emergence of new chemical entities in preclinical stages and ongoing clinical trials.

Omadacycline is an aminomethylcycline that is FDA approved for the treatment of community-acquired bacterial pneumonia (CABP) and acute bacterial skin and skin structure infections (ABSSSI). It stands out among new antibiotics for its advanced stage of development in NTM and its availability in both oral and intravenous formulations (8). Prior research has shown omadacycline’s effectiveness against *Mab* in *in vitro* and animal studies, both when used alone and in combination with other drugs (9–14). Omadacycline presents an attractive alternative to tigecycline, a first-line treatment for *Mab* infection known for causing severe gastrointestinal side effects that limit its use. Data from studies involving healthy volunteers (15) as well as initial trials for CABP (16) and ABSSSI (17) indicate that omadacycline is tolerated better and effectively reaches various lung compartments (18). Based on these and other supportive studies, as well as its high barrier to resistance by *Mab* (13), omadacycline is now being studied as monotherapy for 3 months in a phase 2 trial for patients with *Mab* lung disease (ClinicalTrials.gov identiﬁer NCT04922554). While this trial will provide crucial insights into omadacycline’s activity, tolerability, and efficacy, it will not inform how to combine omadacycline with other drugs to create a complete treatment regimen for patients with *Mab* lung disease.

The goal of this study was to determine the most effective combinations comprising omadacycline for treating lung disease caused by *Mab* isolates seen in the clinic. Building upon previous findings that highlighted the potent activity of two triple-drug combinations—omadacycline + amikacin + imipenem and omadacycline + clofazimine + linezolid—against the *Mab* laboratory reference strain ATCC 19977 (12, 13, 19), these same combinations were selected for further evaluation based on their inclusion in guideline-recommended induction therapy for rapid *Mab* elimination (amikacin, imipenem) or their potential to form an all-oral regimen (clofazimine, linezolid) (3–5).

*Mab* ATCC 19977, isolated in 1950 from a knee abscess (20), has long served as the default reference strain for *Mab* due to its historical significance as the sole isolate available through ATCC until recently. However, assessing the efficacy of experimental regimens against relatively more contemporary isolates from lung disease is crucial, as they are more likely to reflect *Mab* strains encountered in clinical settings today.

Therefore, this study aimed to evaluate the effectiveness of two triple-drug regimens against contemporary isolates with drug-resistant patterns commonly observed in clinical practice. Two *Mab* isolates, M9501 and M9507, collected between 2005 and 2015 from patients with lung disease (21) and subspeciated by whole-genome sequencing (22) were considered. These isolates belong to subspecies *abscessus* and possess an intact *erm(41*) gene. Minimum inhibitory concentrations (MICs) of standard-of-care drugs and several additional antibiotics against the isolates have been previously described (12). While M9501 represents strains susceptible to most antibiotics recommended for treating *Mab* lung disease (3–5), M9507 is more representative of drug-resistant isolates commonly encountered in clinical practice, exhibiting high MIC values for amikacin (>256 mg/L) , azithromycin (32 mg/L), and imipenem (24 mg/L) (Table 1) (12). Moreover, both M9501 and M9507 effectively proliferate in mouse lungs and establish chronic lung infections, meeting essential criteria for evaluating experimental treatments in murine models. Additionally, the efficacy of the omadacycline + clofazimine + linezolid regimen was tested over 6 weeks instead of 4 weeks as undertaken previously, aiming to assess its enduring benefits against the clinical isolates. Given the limited laboratory data on the long-term efficacy of all-oral regimens for treating *Mab* lung disease in humans, this extended evaluation period was deemed important to determine the regimen’s sustained effectiveness.

**TABLE 1:** MIC values of drugs assessed in this study against *M. abscessus* isolates M9501 and M9507^*a*^

Drug	MIC (mg/L) against isolate	
	M9501	M9507
Omadacycline	0.25	0.50
Amikacin	16	>**256**
Imipenem	16	24
Clofazimine	0.25	0.19
Linezolid	**32**	**64**

^*a*^ The MIC values shown here were reported in a prior publication from our group (12). MIC values that are resistant or intermediate according to CLSI breakpoints are denoted in bold or underlined, respectively.

## RESULTS

The regimen omadacycline + amikacin + imipenem was administered for 4 weeks, and its efficacy against the clinical isolates M9501 and M9507 was assessed by enumerating lung *Mab* burden at the end of 1, 2, and 4 weeks of treatment. The regimen omadacycline + clofazimine + linezolid was administered for 6 weeks, and its efficacy against the two isolates was assessed by enumerating lung *Mab* burden at the end of 1, 2, 4, and 6 weeks of treatment. To assess the two test regimens, three additional comparator groups were included. The first group received phosphate-buffered saline (PBS), which served as a control comparator for growth of *Mab* in mouse lungs without antibiotic exposure, as PBS was the solvent used to prepare the antibiotics administered to mice. The second group received omadacycline alone, while the third group received the respective two companion drugs of omadacycline. Early bactericidal activity of a treatment is defined as its ability to reduce *Mab* burden in the lungs of mice during the first 2 weeks of the treatment period, which is in accordance with its use in determining the efficacy of antibacterial treatments in mouse model studies against *Mab* lung infection (13) as well as in humans (23).

**Fig 1:**
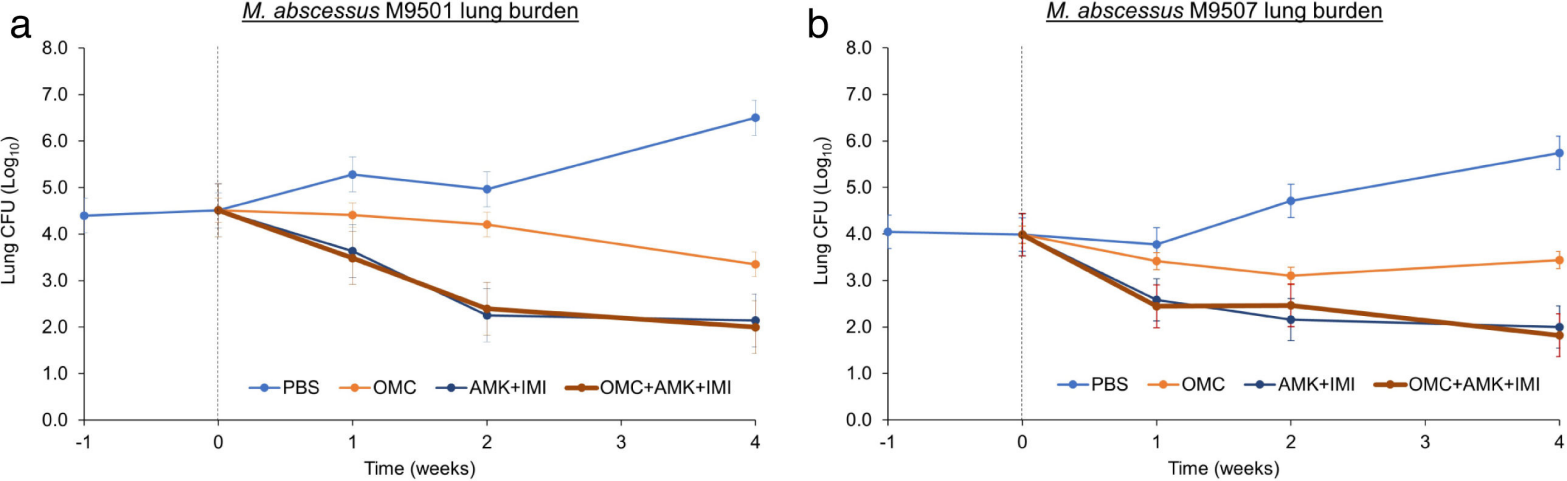
Burdens of *M. abscessus* isolates M9501 (**a**) and M9507 (**b**) in the lungs of C3HeB/FeJ mice treated with triple-drug regimen omadacycline +amikacin + imipenem with comparator groups are shown. Mean lung CFU ± standard error of the mean at 1 week prior to treatment initiation (week −1), on the day of treatment initiation (week 0), and at the completion of 1, 2, and 4 weeks of treatment is shown. *n* = 5 per group at timepoints of weeks −1, 0, 1, and 2, and *n* = 10 per group at week 4. OMC, omadacycline, 15 mg/kg, once daily; AMK, amikacin, 150 mg/kg, once daily; IMI, imipenem, 100 mg/kg/dose, twice daily. Pairwise statistical comparison between each group at each timepoint is included in Table S1.

**Fig 2:**
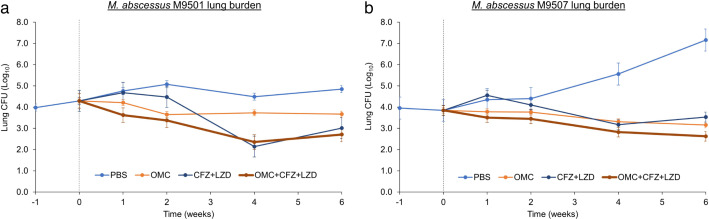
Burdens of *M. abscessus* isolates M9501 (**a**) and M9507 (**b**) in the lungs of C3HeB/FeJ mice treated with triple-drug regimen omadacycline + clofazimine + linezolid with comparator groups are shown. Mean lung CFU ± standard error of the mean at 1 week prior to treatment initiation (week −1), on the day of treatment initiation (week 0), and at the completion of 1, 2, 4, and 6 weeks of treatment is shown. *n* = 5 per group at timepoints of weeks −1, 0, 1, 2, and 4, and *n* = 10 per group at week 6. OMC, omadacycline, 15 mg/kg, once daily; CFZ, clofazimine, 25 mg/kg, once daily; LZD, linezolid, 100 mg/kg, once daily. Pairwise statistical comparison between each group at each timepoint is included in Table S1.

Implantations of M9501 and M9507 in the lungs of mice were comparable, with 4.0–4.5 log_10_ mean CFU per mouse lung recovered 1 day after infection (Fig. 1 and 2). In mice that received only PBS, the lung burden of both isolates increased gradually throughout the study, demonstrating the model’s ability to mimic chronic infection. These mice exhibited signs of illness over time, such as ruffled fur, hunched backs, and lethargy.

In mice treated with omadacycline alone, a modest reduction in the burden of M9501 and M9507 in the lungs was observed over the treatment period. Specifically, omadacycline reduced the burden of M9501 by 1.2 log_10_ and 0.6 log_10_ CFU after 4 weeks of treatment in the two assessments (Fig. 1a and 2a). Similarly, omadacycline reduced the burden of M9507 by 0.54 log_10_ CFU after 4 weeks of treatment in both assessments (Fig. 1b and 2b).

In mice treated with the amikacin + imipenem regimen, the lung burden of M9501 decreased by 2.4 log_10_ CFU after 4 weeks of treatment (Fig. 1a). In comparison, in mice receiving omadacycline + amikacin + imipenem, the reduction in M9501 lung burden was 2.5 log_10_ CFU. Similarly, the amikacin + imipenem regimen reduced M9507 lung burden by 2.0 log_10_ CFU, while the omadacycline + amikacin + imipenem regimen led to a reduction of 2.2 log_10_ CFU for M9507 (Fig. 1b). The addition of omadacycline to amikacin + imipenem did not yield a significant reduction in M9501 or M9507 lung burdens over the 4-week treatment period. Moreover, the efficacies of amikacin + imipenem or omadacycline + amikacin + imipenem against the two isolates were comparable, despite variations in the MICs of amikacin (16 mg/L for M9501 and >256 mg/L for M9507) and imipenem (16 mg/L for M9501 and 24 mg/L for M9507) (Table 1).

In mice treated with the clofazimine + linezolid regimen, there was an increase in M9501 lung burden during the first 2 weeks, indicating a lack of early bactericidal activity of this regimen (Fig. 2a). However, when omadacycline was added to the regimen, it resulted in a reduction in M9501 lung burden from the onset of treatment, demonstrating early bactericidal activity. By the end of the 6-week treatment period, clofazimine + linezolid and omadacycline + clofazimine + linezolid regimens reduced M9501 lung burdens by 1.3 log_10_ and 1.6 log_10_ CFU, respectively (Fig. 2a) (*P* = 0.09). Similarly, the omadacycline + clofazimine + linezolid regimen exhibited early bactericidal activity against M9507, reducing lung burden from the start of treatment, whereas the clofazimine + linezolid regimen showed no early bactericidal activity against M9507, with a 0.7 log_10_ CFU increase during the first week of treatment (Fig. 2b). By the end of the 6-week treatment, the clofazimine + linezolid and omadacycline + clofazimine + linezolid regimens resulted in net reductions of 0.3 log_10_ and 1.2 log_10_ CFU, respectively, of M9507 in the mouse lungs (*P* = 0.05). This difference could not be attributed to random biological variations and therefore was statistically significant.

## DISCUSSION

The incidence of NTM infections, both pulmonary and extrapulmonary, is on the rise worldwide (24). This increase is likely due to multiple factors, including improved diagnostic awareness and capabilities (25), changes in environmental conditions such as increased water coverage (26), the use of certain rheumatologic treatments (27), and in some regions, the rise in cosmetic procedures with inadequate sterilization practices (28, 29). Despite the growing number of cases, treatment approaches and regimen compositions have largely remained unchanged since 1997 (30). However, there is now growing interest, investment, and opportunities for innovative drug and regimen development. Nonetheless, there is still significant effort needed to determine the optimal drugs, doses, and sequence of regimens to ensure safer and more effective treatment outcomes for patients.

This study highlights that the all-oral omadacycline + clofazimine + linezolid regimen exhibits improved bactericidal activity over 6 weeks of treatment in a murine model of pulmonary *Mab* disease. Notably, this study extends the treatment duration beyond previous research, which typically concluded after 4 weeks. Omadacycline monotherapy demonstrated an initial bacteriostatic effect followed by a modest bactericidal effect against both strains over 4 or 6 weeks, consistent with its known pharmacology. Additionally, mice treated with PBS exhibited clinical signs of disease, including reduced resistance when restrained in a mouse cone for injections, ruffled fur, and a hunched back. In contrast, mice that received omadacycline alone showed an improved clinical response: they resisted being restrained in the mouse cone, had normal fur, and maintained normal back posture. This consistent finding suggests that omadacycline has an anti-*Mab* effect early in therapy that extends beyond the reduction in lung *Mab* burden alone.

The omadacycline + clofazimine + linezolid regimen displayed early bactericidal activity and sustained effectiveness against isolates M9501 and M9507, in comparison to placebo, omadacycline alone, or the clofazimine + linezolid combination. These findings suggest that this all-oral combination may be capable of achieving an early reduction of *Mab* burden, a feat typically requiring parenteral therapy. Given the inherent resistance of *Mab* clinical isolates to most antibiotics and their tendency to develop resistance during treatment, omadacycline stands out as a potential option due to its high resistance barrier for *Mab* (13). *Mab* disease is typically treated initially with intravenous antibiotics, such as imipenem and amikacin, due to their high activity levels in the early treatment phases. This study shows that imipenem + amikacin reduces lung *Mab* burden more than the oral regimen during the first 2 weeks. Although clofazimine + linezolid was less effective initially, the combination of omadacycline + clofazimine + linezolid remained bactericidal throughout the treatment. The convenience of this oral triple-drug regimen suggests it could follow the initial rapid lung burden reduction achieved with imipenem + amikacin in the continuation phase.

Previous research has highlighted a discrepancy between the clinical interpretation of MIC patterns of various drugs against *Mab* and their observable clinical efficacy (12, 31). For instance, while imipenem MICs of 16 and 24 mg/L against M9501 and M9507, respectively, would typically be classified as intermediate, and an amikacin MIC of >256 mg/L against M9507 would be classified as resistant according to CLSI breakpoints (32), both amikacin + imipenem and omadacycline + amikacin + imipenem regimens were effective in reducing bacterial counts throughout the treatment period (Fig. 1a and b). Additionally, a previous study found that an *M. abscessus* isolate, M9530, classified as “resistant” based on its imipenem MIC of 48 mg/L according to CLSI guidelines, responded positively to imipenem treatment in mice (12). Sustained sputum culture conversion rates are low (~42%) for *M. abscessus* lung disease (33), even with prolonged parenteral courses of amikacin and imipenem. Omadacycline has the added benefit of providing intracellular activity; however, further study is needed to establish whether the addition of omadacycline increases the likelihood of more durable cure.

The MICs of clofazimine were 0.25 and 0.188 mg/L against M9501 and M9507, respectively, while linezolid MICs fell within the range considered resistant (32 and 64 mg/L for M9501 and M9507, respectively), consistent with clinical observations. Initially, there was a notable increase in *Mab* CFU during the first 1–2 weeks of clofazimine + linezolid administration; however, this regimen subsequently led to a reduction in *Mab* burden. This delayed effect of clofazimine has been observed in previous murine models of both *Mab* and *M. tuberculosis* lung diseases (34, 35).

Our study has limitations in its generalizability. One such limitation is that we assessed drug and regimen efficacy against two clinical isolates only, despite the high heterogeneity of *Mab* strains encountered in clinical settings with variable genotypes and drug sensitivity/resistance phenotypes. Both M9501 and M9507 are from the *abscessus* subspecies. While *abscessus* subspecies is the most prevalent among *Mab*, our study cannot infer generalizability of findings to *Mab* isolates belonging to the *massiliense* and *bolletii* subspecies. While inclusion of additional isolates representing all subspecies would have improved the generalizability of our findings, the larger cohort of mice required for such an approach was beyond the scope of this study. Our primary aim was to provide clear insights into the efficacy of the two experimental regimens involving omadacycline and disseminate relevant information promptly. Therefore, we focused our study on two *Mab* subspecies *abscessus* isolates with distinct drug susceptibility phenotypes.

This study did not include pharmacokinetics (PK) assessment; however, previous research has shown concentration-dependent activity of omadacycline against five distinct *Mab* clinical isolates in time-kill studies (12). Although some variability in killing may have been due to differences in individual omadacycline exposure, prior PK studies demonstrated a dose-proportional increase in exposure *in vivo* as well as exposure-dependent killing *in vitro*. While this study contributes to our collective understanding by extending the treatment duration to 6 weeks, it was not designed to provide insights into the duration needed for relapse-free cure. Nevertheless, this proof-of-principle study justifies future animal studies of longer durations to investigate how these drugs and regimens can be optimally combined and sequenced.

Overall, this study informs both clinicians and clinical trialists as they work together to establish optimal induction and consolidation regimens for treating *Mab* and other NTM infections. The evidence from this study further supports the potential use of omadacycline as part of a combination regimen for treating *Mab* infections. Although the all-oral regimen omadacycline + clofazimine + linezolid was not as effective in killing bacteria as omadacycline + amikacin + imipenem in this model, it still demonstrated bactericidal activity against both *Mab* isolates over 6 weeks. This suggests that for patients who are unable to receive or tolerate the standard parenteral regimen, an all-oral regimen may still provide a significant early *Mab* elimination during treatment. Additionally, for those who complete the initial parenteral treatment phase, this all-oral regimen could be a viable option for consolidation therapy to complete treatment. Further research, including serial testing of these regimens in patients with *Mab* disease, is warranted.

## MATERIALS AND METHODS

### Bacterial strains and *in vitro* growth conditions

Two *Mab* clinical isolates M9501 and M9507, obtained during 2005–2015 from patients with *Mab* lung disease, were selected for this study (21). They belong to the *abscessus* subspecies (22). The MICs of omadacycline against M9501 and M9507 are 0.25 and 0.5 mg/L, respectively (12). The isolates were grown in Middlebrook 7H9 broth (Difco; 271310) supplemented with 0.5% glycerol, 10% albumin-dextrose-saline (ADS) enrichment, and 0.05% Tween-80, as described (36), by incubating at 37°C under constant shaking at 220 rpm. PBS refers to sterile 1× phosphate-buffered saline, pH 7.4 (Quality Biologicals; 114-058-101). To recover *Mab* from mouse lungs, appropriate 10-fold serial dilutions of lung homogenates prepared in PBS, pH 7.4, were inoculated onto Middlebrook 7H11 selective agar (Difco; 283810) supplemented with final 0.5% glycerol, 10% ADS, 50 mg/L carbenicillin (Fisher; 50-213-247), and 50 mg/L cycloheximide (Sigma-Aldrich; C7698) and incubated at 37°C for 5 days.

### Mice, infection, and efficacy studies

C3HeB/FeJ mice (female, 5–6 weeks old) were procured from Jackson Laboratories (Bar Harbor, ME). Four cohorts of mice were procured at different times, but identical infection and treatment protocols were used. As described in the protocol for a mouse model of pulmonary *Mab* disease (37), 100 µL of 1.25 mg/mL solution of dexamethasone prepared in PBS was administered to each mouse once daily beginning 1 week prior to infection with *Mab* and continued throughout the study. This dexamethasone dose is equivalent to 5 mg/kg, as the mean body mass of the mice at 6–8 weeks was ~25 g. To prepare dexamethasone solution, the amount of dexamethasone powder (Sigma-Aldrich; D1756) necessary to administer to all mice in each day was weighed at the beginning of each study, stored in 25 mL polypropylene tube at −20°C, one tube was retrieved on the day of administration, and 1× PBS, pH 7.4, was added and vortexed at high speed for 2 min to prepare a 1.25 mg/mL dexamethasone solution.

Ninety mice per *Mab* isolate were included to test the efficacy of the omadacycline + amikacin + imipenem regimen. Mice were placed in the housing chamber in Glas-Col Inhalation Exposure System (Glas-Col, Terre Haute, Indiana) and simultaneously exposed to aerosol generated from 10 mL of *Mab* suspension at an optical density (A_600nm_) of 0.1 prepared by diluting a culture at logarithmic phase in Middlebrook 7H9 broth. Following 1:1,000 (vol/vol) inoculation in Middlebrook 7H9 broth, M9501 and M9507 typically reach logarithmic phase (A_600nm_ ~0.8–1.2) during 42–48 hours when incubated at 37°C with constant shaking at 220 rpm in an orbital shaker. The infection was carried out in an inhalation exposure system according to manufacturer guidelines (Glas-Col, Terre Haute, Indiana), which included loading of 10 mL of *Mab* suspension in a nebulizer in the exposure system and running the following cycles which the equipment undertakes in an automated manner: preheating for 15 min, aerosol nebulization for 30 min, cloud decay for 30 min, and decontamination for 15 min. Five mice were sacrificed 1 day after infection to determine *Mab* that implanted in the lungs of mice. One week following infection, treatment administration was initiated. On this day, five mice were sacrificed to determine lung *Mab* burden at the onset of treatment administration. Eighty mice that remained were randomly allocated into four groups, 20 mice per group. One group, designated negative control comparator, was treated with PBS, pH 7.4. The second group was treated with omadacycline, the third group with amikacin + imipenem, and the fourth group with omadacycline + amikacin + imipenem. Five mice per treatment group were sacrificed at the completion of 1 and 2 weeks of treatment. Ten mice per treatment group were sacrificed at the completion of 4 weeks of treatment. Lungs were obtained, homogenized, and appropriate dilutions were inoculated onto Middlebrook 7H11 selective agar, incubated for at 37°C for 5 days, and CFU was enumerated.

The efficacy of the omadacycline + clofazimine + linezolid regimen was assessed similarly against isolates M9501 and M9507, but the treatment duration was extended to 6 weeks. A total of 110 mice were used per *Mab* isolate. Five mice per treatment group were sacrificed at the completion of 1, 2, and 4 weeks of treatment. Ten mice per treatment group were sacrificed at the completion of 6 weeks of treatment.

### Antibiotics preparation and administration

Omadacycline was provided by Paratek Pharmaceuticals. Clofazimine was procured from Sigma-Aldrich (C8895). Pharmaceutical-grade powders amikacin (CAS # 39831-55-5), imipenem (CAS # 74431-23-5), and linezolid (CAS # 165800-03-3) were procured from Octagon Chemicals Ltd. Antibiotic preparations were made in PBS, 0.05% agarose solution, or 0.5% carboxymethylcellulose solution as described (19).

Omadacycline, amikacin, clofazimine, linezolid, and PBS were administered once daily. Imipenem was administered twice daily. PBS was administered once daily via oral gavage as 0.2 mL bolus. Omadacycline was administered to mice using a 3.75 mg/mL solution. At the beginning of each treatment week, powder omadacycline was dissolved in PBS to prepare the amount necessary for the week. The amount required for each day was aliquoted into tubes and stored at −20°C. Each day a tube was thawed, and 0.1 mL bolus was administered by subcutaneous injection into the hind dorsal flank to deliver a 15 mg/kg dose per day. This dose is equivalent to 300 mg oral dose in humans (12) as omadacycline lacks oral bioavailability in mice. To administer clofazimine to mice, a 3.125 mg/mL suspension was used. The amount of powder clofazimine necessary for each week was weighed into a 50-mL polypropylene tube, and 0.05% agarose in PBS was added and vortexed at high speed for 5 min to prepare a 3.125 mg/mL suspension. A 0.2 mL bolus of this suspension was administered to each mouse by oral gavage to deliver a 25 mg/kg dose per day. To administer amikacin, a 37.5 mg/mL solution was prepared by dissolving powder amikacin in PBS by sonicating for 10 s with Sonic Dismembrator (Fisher Scientific, Model 100) set at 50% power. A 0.1 mL bolus of this solution was administered by subcutaneous injection into the hind dorsal flank to deliver a 150 mg/kg dose per day. To administer imipenem, the amount for each dosing schedule was weighed into sterile polypropylene tube and stored at −20°C. Each day, an aliquot was retrieved prior to treating mice (morning and evening) and dissolved in PBS by sonicating for 10 s to prepare a 12.5 mg/mL solution. A 0.2 mL bolus of this solution was administered to each mouse by subcutaneous injection into the hind dorsal flank to deliver a 100 mg/kg dose per injection q12 (200 mg/kg per day). Similarly, for linezolid, the amount necessary for each day was aliquoted into a tube, and each day 0.5% carboxymethylcellulose in PBS was added and vortexed to prepare a 12.5 mg/mL suspension. A 0.2 mL bolus of this suspension was administered to each mouse by oral gavage to deliver a 100 mg/kg dose per day.

### Data analysis

Raw lung CFU data were analyzed, and mean ± standard error was determined at each timepoint in each group and graphed using GraphPad Prism v8.4.3. To determine variance between each treatment group at each timepoint, a two-tailed test was used (Table S1), and significance was determined at 95% confidence intervals. The variance that yielded *P* ≤ 0.05 was considered a non-random event, and therefore, the differences in CFU burden between groups were considered significant (* represents *P* ≤ 0.05; ** represents *P* ≤ 0.01), and *P* > 0.05 was considered not significant (represented as “ns”).

## SUPPLEMENTAL MATERIAL

Table S1 msphere.00381-24-s0001.pdf Statistical results of lung Mab burden.

ASM does not own the copyrights to Supplemental Material that may be linked to, or accessed through, an article. The authors have granted ASM a non-exclusive, world-wide license to publish the Supplemental Material files. Please contact the corresponding author directly for reuse.
