# Supplementary material for: Efficacies of omadacycline + amikacin + imipenem and an all-oral regimen omadacycline + clofazimine + linezolid in a mouse model of M. abscessus lung disease
Source: mSphere. 2024 Jul 9;9(7):e00381-24. doi: 10.1128/msphere.00381-24 (PMC11288010; doi:10.1128/msphere.00381-24)
Supplement: Table S1 — Statistical results of lung Mab burden. [file msphere.00381-24-s0001.pdf]

Efficacies of omadacycline+amikacin+imipenem and an all-oral regimen omadacycline+clofazimine+linezolid in a mouse model of *M. abscessus* lung disease

Elisa H. Ignatius[1,2,3], Binayak Rimal[1], Chandra M. Panthi[1], Daniel C. Belz[2,4], Christopher K. Lippincott[1,2], Daniel H. Deck[5], Alisa W. Serio[5], Gyanu Lamichhane[1,2,*]

SUPPLEMENTAL MATERIAL

SUPPLEMENTAL TABLES

Mab isolate ID	Panel of Figure in the manuscript- and drug regimen	PBS or single antibiotic versus antibiotics in combination	*p*-value for variance of the mean lung *Mab* burden at the timepoints specified between treatments groups specified in column 3: Two-Sample Assuming Unequal Variances (P(T<=t) two-tail)			
			Week+1	Week+2	Week+4	Week+6
M9501	Figure 1a OMC+AMK+IMI	PBS vs OMC	0.18	0.11	0.0000005	nd
		PBS vs AMK+IMI	0.03	0.0007	0.00000000001	nd
		PBS vs OMC+AMK+IMI	0.02	0.0005	0.00000000001	nd
		OMC vs AMK+IMI	0.10	0.0001	0.005	nd
		OMC vs OMC+AMK+IMI	0.10	0.0003	0.002	nd
		AMK+IMI vs OMC+AMK+IMI	0.72	0.59	0.52	nd
M9507	Figure 1b OMC+AMK+IMI	PBS vs OMC	0.03	0.05	0.000001	nd
		PBS vs AMK+IMI	0.01	0.01	0.000000004	nd
		PBS vs OMC+AMK+IMI	0.0003	0.02	0.00000005	nd
		OMC vs AMK+IMI	0.04	0.03	0.000002	nd
		OMC vs OMC+AMK+IMI	0.002	0.11	0.0000004	nd
		AMK+IMI vs OMC+AMK+IMI	0.69	0.18	0.28	nd
M9501	Figure 2a OMC+CFZ+LZD	PBS vs OMC	0.37	0.03	0.38	0.0005
		PBS vs CFZ+LZD	0.91	0.24	0.03	0.000002
		PBS vs OMC+CFZ+LZD	0.16	0.04	0.04	0.0000002
		OMC vs CFZ+LZD	0.47	0.13	0.01	0.008
		OMC vs OMC+CFZ+LZD	0.37	0.70	0.01	0.001
		CFZ+LZD vs OMC+CFZ+LZD	0.20	0.13	0.65	0.09
M9507	Figure 2b OMC+CFZ+LZD	PBS vs OMC	0.35	0.36	0.00002	0.0000000000007
		PBS vs CFZ+LZD	0.81	0.66	0.005	0.0008
		PBS vs OMC+CFZ+LZD	0.20	0.20	0.0007	0.000000005
		OMC vs CFZ+LZD	0.27	0.24	0.79	0.63
		OMC vs OMC+CFZ+LZD	0.38	0.27	0.29	0.07
		CFZ+LZD vs OMC+CFZ+LZD	0.17	0.05	0.58	0.05

Table S1: Statistical assessment of *M. abscessus* burden in the lungs of mice receiving different treatments. Results of two tailed t-test of lung *M. abscessus* burdens in different treatment groups at 1-, 2-, 4- and 6-week timepoints are shown. The mean *M. abscessus* lung burdens at 1-, 2-, 4- and 6-weeks of treatment completion timepoints are shown in Figures 1 and 2 of the manuscript. Column 2 lists the three-drug regimens tested and the corresponding figure panel in the manuscript. Column 3 lists the comparisons between all treatment groups within the study; phosphate-buffered-saline (PBS), omadacycline (OMC), imipenem (IMI), linezolid (LZD), amikacin (AMK), and clofazimine (CFZ). Column 4 lists *p*-values for variance of the mean lung *Mab* burden at the completion of one week of treatment between treatment groups shown in the corresponding row in column 3. Similarly, columns 5, 6 and 7 list *p*-values for variance of the mean lung *Mab* burden at the completion of two-, four- and six weeks of treatment, respectively, between treatment groups shown in the corresponding row in column 3. 'nd' represents 'not determined' as evaluation of efficacy of OMC+AMK+IMI were undertaken for four weeks.
